# Supplementary material for: Single duplex DNA sequencing with CODEC detects mutations with high sensitivity
Source: Nat Genet. 2023 Apr 27;55(5):871–9. doi: 10.1038/s41588-023-01376-0 (PMC10181940; doi:10.1038/s41588-023-01376-0)
Supplement: Supplementary file 1 — Supplementary Text and Supplementary Tables 2–6. [file 41588_2023_1376_MOESM1_ESM.pdf]

nature genetics

Technical Report https://doi.org/10.1038/s41588-023-01376-0

Single duplex DNA sequencing with CODEC detects mutations with high sensitivity

In the format provided by the authors and unedited

Supplementary Text

CODECSuite: demultiplexing

CODEC sequencing reads start with Unique Molecular Identifier (UMI) sequences: NNN or NNNA or NNNT (NNN is a random 3-mer), followed by an 18 bp sample barcode and a T base (Extended Data Figure 1f). To demultiplex, CODECSuite extracts the barcode (4th - 21st bases from the 5′-end) and uses smith-waterman (SW) algorithm [1] for sample indices (SID) assignments. If the extracted barcode is within x edit distance (default 3) away from one and only one sample index, it is declared as a match. Then, a read pair is successfully demultiplexed if and only if the two extracted barcodes (one from each end of the read pair) both match the expected SID (P5 and P7). Only successfully demultiplexed reads are used for subsequent steps and the expected SID are stored in the read names for the subsequent adapter trimming step. Besides, when the two barcodes from a read pair match a chimeric sample index combination, CODECsuite also checks index hopping by aligning the two inserts and flags them as hopping reads if they overlap. Otherwise, the mixed indices are most likely a result of intermolecular byproduct.

CODECSuite: adapter trimming and byproducts cleaning

The demultiplexing step adds SID to the read name but does not alter the read sequencing. The adapter trimming step removes the adapter sequences from the read and output as uBAM (unmapped BAM format). The first 3 bases of R1 and R2 are cut and hyphenated and added to the 'RX' tag in the bam record. Each correct CODEC read contains a 5′ adapter and a possible 3′ adapter (in sequencing orientation). The R1's SID is used as the template to trim the R1's 5′ adapter and the reverse complement of R2's SID is used to trim R1's 3′ adapter, and vice versa for trimming R2. Again, SW algorithm is used to find a match. We group the reads based on if the 5′ adapter is found on both R1 and R2. In other words, only read pairs with 5′ adapters found in both reads (successfully passing the demultiplexing step) were kept for analyses in this study and be counted towards the 'Read pairs' column in the Supplementary Table 1. We often observed around 70% of the reads successfully passing the de-multiplex step. One of the potential explanations is the low clustering efficiency of long library molecules. Note that CODEC byproducts were also counted toward the 'Read pair' column. To distinguish CODEC byproducts vs. correct products, we checked the 3′ adapter sequence, if it existed. If the 3′ adapter sequence was found but the insert length was too short (e.g., < 15 bp), we discarded the read. If both R1 and R2 were discarded, the template was considered as a blank ligation. If only one of the read ends was discarded, it was classified as a double ligation. Lastly, we checked if R1 and R2 were mapped with expected distance (e.g., < 500 bp) and orientation to check intermolecular byproducts. The 'Correct product coverage' column in Supplementary Table 1 was calculated after the byproducts were removed. The summary of byproducts formation and quantification was made by a custom python script also available at the CODECsuite github site.

CODECSuite: read pairs and duplex consensus

CODECsuite can generate de novo or reference-based consensus. The reference-based consensus has better accuracy and is used throughout this study. A consensus base is formed if two aligned bases (or gaps in terms of insertion or deletion) agree and N otherwise. CODECsuite keeps the pair-end reads but replaces the read sequence with consensus sequence for both R1 and R2. The sequence quality and other auxiliary tags such as UMI are kept intact. The consensus is generated at uBAM format.

CODECSuite: CODEC-MSI

CODEC-MSI first scans for homopolymer sites where there is at least one read having ≥2 bp deletion for a homopolymer length of ≤18 or ≥3 bp deletion for length >18 and all reads are reference type in the normal. It jointly models tumor and normal to account for the shallow depth (e.g., a heterozygous germline indel is missing in the normal because of low depth). Several other features of the model also help to alleviate this problem. We include tumor purity in the model as a nuisance parameter which is first learned across all loci of interest. This helps to filter germline sites when the tumor purity is low. We also use a database VCF (such as gnomAD) as a genotype prior to

account for common germline indels and noisy sites. We assume each read is observed independently and use the empirical error rate observed in deep CODEC WGS NA12878 to calculate the likelihood of each observed homopolymer length given the reference length. The sum of per-site MSI posterior probability is used as the sample MSI score and was plotted in Figure 5c.

CODEC-MSI outputs a sample-level MSI score by summing over of the posterior probability (a.k.a. genotype quality (GQ) score) of all MSI-positive loci. Thereby, CODEC-MSI model is essentially an indel (more specifically Short Tandem Repeat (STR)) calling model. By jointly modeling the tumor and normal samples and limiting the genotype to be only AA = Homozygous reference or AB = Heterozygous, it computes the posterior probability of a MS locus being:

$$P(\mathrm{G} = \{\mathrm{AA}, \mathrm{AB}\}|\mathrm{D}) = \frac{P(\mathrm{D}|\mathrm{G})\, P(\mathrm{G})}{\sum_{\mathrm{G}} P(\mathrm{D}|\mathrm{G})\, P(\mathrm{G})}.$$

Without subscript, G = $\{G_s, G_g\}$ is the combined genotype of somatic and germline mutations; G_s or $G_g \in \{AA, AB\}$. D stands for the data, L is the vector of repeat lengths (e.g., len(ATATAT) = 3) for a AT repeat) in tumor, and y is the number of reference fragments in normal. And we ignore a site if the normal contains any non-reference reads. The likelihood function $P(D|G) = P(L|G_s, G_g)\, P(y|G_g)$ assumes independence of tumor and normal sample. Here, the $P(y|G_g)$ is a binomial distribution with $p = 0.5$ for AB and $p = 0.999$ (assuming error rate 10^{-3}) for AA. Since when depth is low, we may not resolve the genotype. However, the larger the y, the more likely that G_g = AA. Similar to Saunders et al. [2], we modeled tumor sample as a mixture of the normal sample with somatic mutations and normal sample as a mixture of germline mutations with noise. In tumor sample, each fragment is independently observed thus have

$$P(L|\mathrm{G_s}, \mathrm{G_g}) = \prod_{I} P(l_i|\mathrm{G_g})\, P(\mathrm{T}_i = \mathrm{g}) + P(l_\mathrm{i}|\mathrm{G_S})\, P(\mathrm{T}_i = \mathrm{s}).$$

Here, $P(T_i = g)$ is the probability of read i originating from a germline cell and $P(T_i = s)$, a somatic cell. Note that $P(T_i = g) + P(T_i = s) = 1$ so that we can reduce to a scalar parameter η. Strictly speaking, η should vary locus by locus according to the clonalities. Practically, this is very hard to estimate given the low depth (<4x). Hence, we let η represent tumor fraction which we can estimate using all informative loci. Next, for each read pair, we assume it is generated by one chromosome (or a haplotype) which is again randomly selected with $p = 0.5$ from a diplotype. We estimate the alternative haplotype from the tumor sample by taking the most probable non-reference haplotype, i.e., the one has the greatest number of read support. We calculate the diplotype to read likelihood by summing over the two possible haplotypes:

$$P(L_i|\mathrm{G}) = \sum_{H \in \{h_1, h_2\}} P(l_i|H)\, P(H|\mathrm{G})$$

where $P(l_i|H)$ is the empirical distribution, in this case the observed repeat length distribution conditional on the true repeat length. We used CODEC on NA12878 to estimate the repeat length empirical distribution, represented by the heatmap in Figure 5b. To account for the low depth and potential contamination, we utilize public variant database such as gnomAD and use the population variant allele frequency in the prior probability: $P(G) = P(N)\, P(S)$, where $P(N)$ and $P(S)$ are Bernoulli distribution with $p_n = \max(\mathrm{popAF}, 10^{-4})$ and $p_s = 0.5$, when genotype is AB.

Duplex recovery and downsampling to certain family sizes

Custom python scripts were used to downsample in Figure 2b,c and 4i. For duplex recovery and mutant duplex recovery, we subsampled the pre-consensus family-assigned reads (after Fgbio GroupReadsByUmi) per target (Figure 2c or whole-genome-wide (Figure 4i) at log spaced fractions starting from 10-4 (np.logspace(-4, 0, 30)) and calculated the number of duplexes or Mutect2-supported mutant duplexes formed at each downsampled fraction, respectively. In this study, this allowed us to understand situations when only limited sequencing was given (e.g., <100 read pairs). To understand the impact of family size on residual SNV rate, we wrote another python script for downsampling (Figure 2b). In our sample, the number of duplex consensuses having the exact family sizes (number of pre-collapsed

raw read pairs) was limited and thus gave less confident results. Thus, we took advantage of families with strictly larger family sizes and downsampled them to have certain target family size. We also sought to maintain an equal or close ratio between the number of reads from each strand.

WGS coverage calculation and cost comparison

On WGS data from CODEC and standard NGS, we first used 'Picard MarkDuplicates' for deduplication. Then, 'Picard CollectWgsMetrics' with default parameters was used to calculate the coverage. The coverage of CODEC WGS considered all CODEC reads including byproducts. When calculating CODEC correct product coverage, we first removed byproducts (discordant reads) using SAMtools and then ran 'Picard CollectWgsMetrics'. Duplex depth is the number of final duplex bases (passing all CODEC filters) divided by the size of interrogated genomic regions. The numbers of final duplex bases were calculated by single-fragment mutation caller and used as the denominator for calculating residual mutation rates. The numbers of final duplex bases were also used for calculating the cost of CODEC vs. Duplex Sequencing in Figure 2d. The cost of CODEC and Duplex Sequencing were proportional to the number of raw read pairs (CODEC 214M, Duplex Sequencing 305M). The actual cost of Illumina sequencing is assumed at $7/Gb (assuming NovaSeq 6000 S4 and accounting for the Q30 rate).

Fragment-level and base-level filters

1) The number of Q30+Q30 bases (bases from R1 and R2 are both equal to or above Q30) is at least 70% of the overlap length of R1 and R2. 2) The number of discordant bases between R1 and R2 and non-ACGT bases is less than 5% of the total read length. 3) Mapping quality is at least 60. 4) No 5′ end clipping is allowed. 5) Fragment length is above 30 bp. 6) The secondary alignment score is no more than 50% of the primary alignment score. 7) Each read is allowed to have less than 5 non-N substitutions (base from the read is A/C/G/T and is different from the reference genome). 8) No cluster of mutations near the fragment ends, i.e., three mutations are spaced out less than 15 bp from each other and from either end of the fragment. 9) A read should contain no indel when it is used for calling SNVs.

Apart from the fragment-level filters, we have a few base-level filters: 1) both bases from R1 and R2 should have quality scores equal to or above 30 (≥Q30) and should agree with each other, 2) the bases immediately preceding or succeeding of indel should be ≥Q30 and are not N, 3) mutations within 12 bp from the fragment ends are not called, and 4) 95% of the read-pairs in the same family should agree on the mutation that is being called.

Comparing to standard NGS on single-fragment mutation calling

When comparing CODEC WGS vs. standard WGS on detecting mutations from single DNA molecules, we first matched on their coverage. GATK DownsampleSam was used in all the scenarios. For the eight breast cancer tumors used in Figure 4a,g,h, we downsampled the standard WGS tumor samples to match CODEC coverage on per sample basis (range 1.37x to 2.38x). The 15 normal buffy-coat standard NGS samples used in Figure 3c were downsampled to 6x since CODEC coverage has a tighter distribution (5.46x to 6.93x). Note that two of the CODEC samples originally had high coverage 18x and we downsampled them to 5.6x. Lastly, to generate Figure 4e, we downsampled the standard WGS MSI-H tumors from 12x to 1x, with an incremental of 1, and CODEC from 5x to 3x, 1x, 0.5x, 0.25x, 0.125x, 0.05x, and finally to 0.025x. CODEC single-fragment mutation caller was used by the standard NGS data in these scenarios with a couple of slight modifications: 1) We allowed mutations called from the overhang regions where R1 and R2 do not overlap since the fragment sizes of the standard NGS are usually larger. 2) the Q30 + Q30 filter was changed to calculating the ratio of Q30 bases throughout the read pair, again because of the small or 0 overlap between R1 and R2 in standard NGS. Similar to duplex depth of CODEC data, we thus can also calculate the number of final evaluated bases from standard NGS (Supplementary Table 1).

Examining clonal tumor mutation detection with CODEC WGS

We deeply sequenced a tumor biopsy (Figure 4b) (tumor purity = 0.86) using CODEC with 509M raw read pairs. The purity was estimated by ABSOLUTE [3] on 60x standard WGS. We downsampled the data to 10%, 20%,…, 90% of total reads and called mutations using the single-fragment mutation caller. To strike a balance between sensitivity and specificity, we chose to use a Q30+Q30 cutoff at 50%. The rest of the filters were kept the same as described in the

earlier section. From all somatic SNVs called from 60x WGS by Mutect2, we selected a set of SNVs (n = 3,408) with cancer cell fraction (CCF, estimated by ABSOLUTE) ≥ 0.9 and within our high-complexity regions (2.3B) as clonal SNVs. When estimating the PPV, we used all SNVs (n = 11,714) as the ground truth without restricting to CCF. For CODEC data, we called SSNVs with at least 1 or 2 mutant duplex support. The theoretical sensitivities were estimated by a binomial distribution with $p = 0.86 \times 0.5$, assuming all heterozygous mutations. The projection was through binomial distributions where p was estimated from the data, which we found to be lower than the ideal case.

Sperm DNA purification

The sperm sample was obtained as part of an IRB-approved human subjects protocol at New York University Grossman School of Medicine. Sperm was purified from semen prior to initial freezing by density gradient centrifugation, and after thawing for processing by a second density gradient centrifugation with ORIGIO gradient 40/80 buffer (Cooper Surgical, 84022060) per the manufacturer's instructions. An aliquot of sperm was then centrifuged at 300 RCF for 5 minutes at room temperature. The supernatant was removed, leaving approximately 50 μL of sperm/buffer at the bottom of the microtube. The tube was tapped gently 5 times to break up the sperm pellet before adding lysis buffer. Sperm lysis buffer was prepared by combining (for each sample) 497.5 μL of QIAGEN Buffer RLT (QIAGEN, 79216) without β-mercaptoethanol, and 2.5 μL of 0.5 M Bond-Breaker TCEP Solution (Thermo Scientific, 77720) for a lysis buffer with 2.5 mM TCEP final concentration. Five hundred microliters of sperm lysis buffer were added to the sperm sample, without pipette mixing. One hundred milligrams of 0.2 mm stainless steel beads (Next Advance, SSB02) were then added to each sample and homogenization was performed with the TissueLyser II (QIAGEN) at 20 Hz. DNA was then extracted using the QIAamp DNA Mini Kit (QIAGEN, 51306) with a modified protocol as follows. Five hundred microliters of buffer AL were added to each lysate and vortexed well. Then, 500 μL of 100% ethanol were added and vortexed well. Then, the mixture was applied to a QIAamp DNA Mini spin column and the remaining standard QIAamp protocol was followed. DNA was eluted with 100 μL of 10 mM Tris pH 8. RNase treatment was then performed by adding 12 μL of 10x PBS pH 7.4 (Gibco, 70011044), 2 μL of Monarch RNase A (NEB, T3018L), and 6 μL nuclease-free water. The reaction was incubated at room temperature for 5 minutes and immediately purified using 0.8x SPRI beads with elution using 35 μL of low TE buffer. A methylation-based somatic cell contamination assay was performed [4] to confirm sperm purity.

Reference

[1] Smith, T. F. & Waterman, M. S. Identification of common molecular subsequences. J. Mol. Biol. 147, 195–197 (1981).

[2] Saunders, C. T. et al. Strelka: accurate somatic small-variant calling from sequenced tumor–normal sample pairs. Bioinformatics 28, 1811–1817 (2012).

[3] Carter, S. L. *et al.* Absolute quantification of somatic DNA alterations in human cancer. *Nat. Biotechnol.* **30**, 413–421 (2012).

[4] Jenkins, T. G., Liu, L., Aston, K. I. & Carrell, D. T. Pre-screening method for somatic cell contamination in human sperm epigenetic studies. *Syst. Biol. Reprod. Med.* **64**, 146–155 (2018).

Supplementary Table 2. Details of each sequencing metric

Metric	Definition	What it represents	Applicability		
			Standard NGS	Duplex Sequencing	CODEC
Raw read pairs	Number of raw read pairs generated by a sequencer	Cost of sequencing	o	o	o
Deduplicated coverage	Mean sequencing coverage after removing duplicate reads	Data without redundancy	o	o	o
Correct product depth	Deduplicated coverage from read pairs with the correct CODEC structure	CODEC data without byproduct reads			o
Duplex depth	Mean depth of sequencing from sequenced bases in consensuses between Watson and Crick strands	Highly accurate data that passed all analytical filters		o	o

Supplementary Table 3. Evaluation of SNP + small indel calls between CODEC WGS and standard WGS. Table were generated by Vcfeval.

Method	Coverage	True-pos-baseline	True-pos-call	False pos	False neg	Precision	Sensitivity	F-measure	FPPM	FNR
CODEC	1	639,183	639,205	8,352	3,051,678	0.987	0.173	0.295	3.2	0.827
	2	1,091,957	1,092,003	13,122	2,598,904	0.988	0.296	0.455	5.1	0.704
	3	1,442,970	1,443,029	16,110	2,247,891	0.989	0.391	0.560	6.2	0.609
	4	1,795,929	1,795,997	18,839	1,894,932	0.990	0.487	0.652	7.3	0.513
	5	2,038,899	2,038,965	20,677	1,651,962	0.990	0.552	0.709	8.0	0.448
	40 (correct product depth=17)	3,471,473	3,471,571	29,748	219,388	0.992	0.941	0.965	11.5	0.057
	40 (utilizing byproducts)	3,592,683	3,592,792	89,747	98,178	0.976	0.973	0.975	34.8	0.026
Standard WGS	1	1,362,228	1,362,280	263,618	2,328,633	0.838	0.369	0.512	102.4	0.631
	2	2,001,169	2,001,242	332,255	1,689,692	0.858	0.542	0.664	129.0	0.458
	3	2,453,841	2,453,932	366,744	1,237,020	0.870	0.665	0.754	142.4	0.335
	4	2,778,411	2,778,517	387,976	912,450	0.878	0.753	0.810	150.7	0.247
	5	2,990,581	2,990,709	392,938	700,280	0.884	0.810	0.846	152.6	0.190
	17	3,626,497	3,626,631	211,471	64,364	0.945	0.983	0.963	82.0	0.017

True-pos-baseline: number of true positives in baseline VCF file

True-pos-call: number of true positives in evaluation VCF file

FPPM: False positive per million bases

FNR: False negative ratio

Supplementary Table 4. Number of targeted sites with SNV detected by CODEC and Duplex Sequencing.

Sample	Targeted	Detected by CODEC	Detected by DupSeq
Patient 1	99	97	94
Patient 3	18	18	12
Patient 5	38	38	36
Patient 9	426	425	425
Total	581	578	567

Supplementary Table 5. Sequences of oligonucleotides. Colors match Figure 1.

	**CODEC adapter**	
LD4-adap5-1	AATGATACGGCGACCACCGAGATCTACACCTTGAACGGACTGTCCAC*T	Set 1
LD4-adap5-2	AATGATACGGCGACCACCGAGATCTACACGAGCCTACTCAGTCAACG*T	
LD4-adap5-3	AATGATACGGCGACCACCGAGATCTACACGCTTGTAAGGCAGGTTAG*T	
LD4-adap5-4	AATGATACGGCGACCACCGAGATCTACACCAAGCGTCTTACATGGTC*T	
LD4-adap5-5	AATGATACGGCGACCACCGAGATCTACACCTGGTCCAAGAACGTCTG*T	Set 2
LD4-adap5-6	AATGATACGGCGACCACCGAGATCTACACGATCCAGTTCTGTCGAGC*T	
LD4-adap5-7	AATGATACGGCGACCACCGAGATCTACACCCTATAGGTGCAACGAAG*T	
LD4-adap5-8	AATGATACGGCGACCACCGAGATCTACACGAAGGTCCACTGTATCTC*T	
LD4-adap7-1	CAAGCAGAAGACGGCATACGAGATCACCGAGCGTTAGACTAC*T	Set 1
LD4-adap7-2	CAAGCAGAAGACGGCATACGAGATGTGTCGAACACTTGACGG*T	
LD4-adap7-3	CAAGCAGAAGACGGCATACGAGATCTGATCTTCAGCTGACTG*T	
LD4-adap7-4	CAAGCAGAAGACGGCATACGAGATGAATCTGAGGCACTGTAC*T	
LD4-adap7-5	CAAGCAGAAGACGGCATACGAGATCTCTGAACGATCGAGCTC*T	Set 2
LD4-adap7-6	CAAGCAGAAGACGGCATACGAGATGAGGTGCATGCACCTTAG*T	
LD4-adap7-7	CAAGCAGAAGACGGCATACGAGATCTAACTTCCATTGCACTC*T	
LD4-adap7-8	CAAGCAGAAGACGGCATACGAGATGACCTGGATGGATAGGAG*T	
LD4-brid5-1	P-GTGGACAGTCCGTTCAAGNNNAGATCGGAAGAGCGTCGTGTAGGGAAAGAGTGTTTACATAGTTATCCGCTAGACTCTGACGTGTTGATCCTCGAAGC	Set 1
LD4-brid5-2	P-CGTTGACTGAGTAGGCTCNNNAGATCGGAAGAGCGTCGTGTAGGGAAAGAGTGTTTACATAGTTATCCGCTAGACTCTGACGTGTTGATCCTCGAAGC	
LD4-brid5-3	P-CTAACCTGCCTTACAAGCTNNNAGATCGGAAGAGCGTCGTGTAGGGAAAGAGTGTTTACATAGTTATCCGCTAGACTCTGACGTGTTGATCCTCGAAGC	
LD4-brid5-4	P-GACCATGTAAGACGCTTGANNNAGATCGGAAGAGCGTCGTGTAGGGAAAGAGTGTTTACATAGTTATCCGCTAGACTCTGACGTGTTGATCCTCGAAGC	
LD4-brid5-5	P-CAGACGTTCTTGGACCAGNNNAGATCGGAAGAGCGTCGTGTAGGGAAAGAGTGTTTACATAGTTATCCGCTAGACTCTGACGTGTTGATCCTCGAAGC	Set 2
LD4-brid5-6	P-GCTCGACAGAACTGGATCNNNAGATCGGAAGAGCGTCGTGTAGGGAAAGAGTGTTTACATAGTTATCCGCTAGACTCTGACGTGTTGATCCTCGAAGC	
LD4-brid5-7	P-CTTCGTTGCACCTATAGGTNNNAGATCGGAAGAGCGTCGTGTAGGGAAAGAGTGTTTACATAGTTATCCGCTAGACTCTGACGTGTTGATCCTCGAAGC	
LD4-brid5-8	P-GAGATACAGTGGACCTTCANNNAGATCGGAAGAGCGTCGTGTAGGGAAAGAGTGTTTACATAGTTATCCGCTAGACTCTGACGTGTTGATCCTCGAAGC	
LD4-brid7-1	P-GTAGTCTAACGCTCGGTGNNNAGATCGGAAGAGCACACGTCTGAACTCCAGTCACCAATCTATAAGTTGCTTCGAGGATCAACACGTCAGAGTCTAGC	Set 1
LD4-brid7-2	P-CCGTCAAGTGTTCGACACNNNAGATCGGAAGAGCACACGTCTGAACTCCAGTCACCAATCTATAAGTTGCTTCGAGGATCAACACGTCAGAGTCTAGC	
LD4-brid7-3	P-CAGTCAGCTGAAGATCAGTNNNAGATCGGAAGAGCACACGTCTGAACTCCAGTCACCAATCTATAAGTTGCTTCGAGGATCAACACGTCAGAGTCTAGC	
LD4-brid7-4	P-GTACAGTGCCTCAGATTCANNNAGATCGGAAGAGCACACGTCTGAACTCCAGTCACCAATCTATAAGTTGCTTCGAGGATCAACACGTCAGAGTCTAGC	
LD4-brid7-5	P-GAGCTCGATCGTTCAGAGNNNAGATCGGAAGAGCACACGTCTGAACTCCAGTCACCAATCTATAAGTTGCTTCGAGGATCAACACGTCAGAGTCTAGC	Set 2
LD4-brid7-6	P-CTAAGGTGCATGCACCTCNNNAGATCGGAAGAGCACACGTCTGAACTCCAGTCACCAATCTATAAGTTGCTTCGAGGATCAACACGTCAGAGTCTAGC	
LD4-brid7-7	P-GAGTGCAATGGAAGTTAGTNNNAGATCGGAAGAGCACACGTCTGAACTCCAGTCACCAATCTATAAGTTGCTTCGAGGATCAACACGTCAGAGTCTAGC	
LD4-brid7-8	P-CTCCTATCCATCCAGGTCANNNAGATCGGAAGAGCACACGTCTGAACTCCAGTCACCAATCTATAAGTTGCTTCGAGGATCAACACGTCAGAGTCTAGC	
	**CODEC blockers for hybridization capture**	
LD4-HybBlk1	AGATCGGAAGAGCGTCGTGTAGGGAAAGAGTGTTTACATAGTTATCCGCTAGACTCTGACGT-3C	
LD4-HybBlk2	AGATCGGAAGAGCACACGTCTGAACTCCAGTCACCAATCTATAAGTTGCTTCGAGGATCAAC-3C	
	**Duplex Sequencing adapter**	
Adapter 1	ACACTCTTTCCCTACACGACGCTCTTCCGATCTNNN*T	
Adapter 2	NNNAGATCGGAAGAGCACACGTCTGAACTCCAGTCAC	
Adapter 3	ACACTCTTTCCCTACACGACGCTCTTCCGATCTNNNN*T	
Adapter 4	NNNNAGATCGGAAGAGCACACGTCTGAACTCCAGTCAC	

"*" indicates phosphorothioate backbone modification. "P-" indicates 5'-phosphorylation. "-3C" indicates C3 spacer.

Supplementary Table 6. Somatic SNVs of patient 315 and a healthy donor detected by IDT pan-cancer panel (800 kb).

HUGO symbol	Chromosome	Start position	Reference allele	Tumor allele	Participant	Sample	VAF	Tumor ref count	Tumor alt count	Normal ref count	Normal alt count
PIK3CA	3	178,936,091	G	C	patient	cfDNA	0.246	43	14	178	0
CDKN2A	9	21,971,000	C	A	patient	cfDNA	0.264	39	14	158	1
ARHGAP35	19	47,507,737	C	T	patient	cfDNA	0.281	46	18	140	0
KMT2C	7	151,927,016	T	C	patient	buffy coat	0.090	NA	NA	NA	NA
KMT2C	7	151,935,831	G	A	patient	buffy coat	0.070	NA	NA	NA	NA
KMT2C	7	151,935,831	G	A	healthy donor	buffy coat	0.040	NA	NA	NA	NA
KMT2C	7	151,970,859	C	T	healthy donor	buffy coat	0.050	NA	NA	NA	NA

Tumor ref count: number of unique duplexes with reference allele in cfDNA data

Tumor alt count: number of unique duplexes with tumor allele in cfDNA data

Normal ref count: number of unique duplexes with reference allele in the matching normal data

Normal alt count: number of unique duplexes with tumor allele in the matching normal data
