# Supplementary material for: Single duplex DNA sequencing with CODEC detects mutations with high sensitivity
Source: Nat Genet. 2023 Apr 27;55(5):871–9. doi: 10.1038/s41588-023-01376-0 (PMC10181940; doi:10.1038/s41588-023-01376-0)
Supplement: Supplementary file 2 — Reporting Summary [file 41588_2023_1376_MOESM2_ESM.pdf]

nature portfolio

Corresponding author(s): Viktor A. Adalsteinsson

Last updated by author(s): Mar 17, 2023

Reporting Summary

Nature Portfolio wishes to improve the reproducibility of the work that we publish. This form provides structure for consistency and transparency in reporting. For further information on Nature Portfolio policies, see our Editorial Policies and the Editorial Policy Checklist.

Statistics

For all statistical analyses, confirm that the following items are present in the figure legend, table legend, main text, or Methods section.

n/a	Confirmed	
☐	☒	The exact sample size (n) for each experimental group/condition, given as a discrete number and unit of measurement
☐	☒	A statement on whether measurements were taken from distinct samples or whether the same sample was measured repeatedly
☐	☒	The statistical test(s) used AND whether they are one- or two-sided<br>*Only common tests should be described solely by name; describe more complex techniques in the Methods section.*
☐	☒	A description of all covariates tested
☐	☒	A description of any assumptions or corrections, such as tests of normality and adjustment for multiple comparisons
☐	☒	A full description of the statistical parameters including central tendency (e.g. means) or other basic estimates (e.g. regression coefficient) AND variation (e.g. standard deviation) or associated estimates of uncertainty (e.g. confidence intervals)
☐	☒	For null hypothesis testing, the test statistic (e.g. F, t, r) with confidence intervals, effect sizes, degrees of freedom and P value noted<br>*Give P values as exact values whenever suitable.*
☐	☒	For Bayesian analysis, information on the choice of priors and Markov chain Monte Carlo settings
☒	☐	For hierarchical and complex designs, identification of the appropriate level for tests and full reporting of outcomes
☐	☒	Estimates of effect sizes (e.g. Cohen's d, Pearson's r), indicating how they were calculated

Our web collection on statistics for biologists contains articles on many of the points above.

Software and code

Policy information about availability of computer code

Data collection: No software was used.

Data analysis:
CODEC data processing: https://doi.org/10.5281/zenodo.7705860
bcl2fastq v2.2
BWA v0.7.17
Samtools v1.15.1
Picard v2.27.1: http://broadinstitute.github.io/picard/
fgbio v2.0.2: https://github.com/fulcrumgenomics/fgbio
GATK for germline: 4.1.4.1
GATK/mutect2 for somatic: 4.1.7.0
DeconstructSigs v1.9.0: https://github.com/raerose0l/deconstructSigs
Breast cancer signatures: https://github.com/Nik-Zainal-Group/signature.tools.lib#examplese01
Sigfit v2.2: https://github.com/kgori/sigfit
Pipeline or workflow management: Snakemake 7.3.8
Plot and data wrangling and statistic analysis: R (v4.1); Python (v3.7); ggplot2 (v3.3.5); tidyverse (v1.3.1); data.table (v1.14.2); Pandas (v1.3.3); Pysam (v0.16); seaborn (v0.11.2)

For manuscripts utilizing custom algorithms or software that are central to the research but not yet described in published literature, software must be made available to editors and reviewers. We strongly encourage code deposition in a community repository (e.g. GitHub). See the Nature Portfolio guidelines for submitting code & software for further information.

Data

Policy information about availability of data

All manuscripts must include a data availability statement. This statement should provide the following information, where applicable:

- Accession codes, unique identifiers, or web links for publicly available datasets
- A description of any restrictions on data availability
- For clinical datasets or third party data, please ensure that the statement adheres to our policy

Depositing DNA sequencing data and results generated for this study such as Mutect2 MAF files to dbGaP is ongoing. NA12878 PacBio data was downloaded from GIAB https://ftp-trace.ncbi.nlm.nih.gov/ReferenceSamples/giab/data/NA12878/PacBio_SequelII_CCS_11kb/.

Field-specific reporting

Please select the one below that is the best fit for your research. If you are not sure, read the appropriate sections before making your selection.

☒ Life sciences ☐ Behavioural & social sciences ☐ Ecological, evolutionary & environmental sciences

For a reference copy of the document with all sections, see nature.com/documents/nr-reporting-summary-flat.pdf

Life sciences study design

All studies must disclose on these points even when the disclosure is negative.

Sample size	All clinical samples were selected based on their availability for this study (>20 ng for buffy coat, 1> ng for tumor DNA, and > 2.5 ng for cell-free DNA).
Data exclusions	In Extended Data Figure 7, a tumor sample with high subclonality was removed.
Replication	All experiments were performed independently and have no replicates.
Randomization	There was no allocating into experimental groups.
Blinding	There was no allocating into experimental groups.

Reporting for specific materials, systems and methods

We require information from authors about some types of materials, experimental systems and methods used in many studies. Here, indicate whether each material, system or method listed is relevant to your study. If you are not sure if a list item applies to your research, read the appropriate section before selecting a response.

Materials & experimental systems

n/a	Involved in the study	
☒	☐	Antibodies
☐	☒	Eukaryotic cell lines
☒	☐	Palaeontology and archaeology
☒	☐	Animals and other organisms
☐	☒	Human research participants
☒	☐	Clinical data
☒	☐	Dual use research of concern

Methods

n/a	Involved in the study	
☒	☐	ChIP-seq
☒	☐	Flow cytometry
☒	☐	MRI-based neuroimaging

Eukaryotic cell lines

Policy information about cell lines

Cell line source(s)	NA12878 (purified DNA purchased from Coriell)
Authentication	Coriell uses multiplexed PCR for 6 autosomal microsatellite markers.
Mycoplasma contamination	Cultures from Coriell are found free of mycoplasma.
Commonly misidentified lines (See ICLAC register)	N/A

Human research participants

Policy information about studies involving human research participants

Population characteristics	Patients from Dana-Farber Cancer Institute protocols 05-246, 13-383, and 05-055 had breast cancer. A pair of colon cancer and adjacent normal colon samples were obtained from the Massachusetts General Hospital Tissue Bank. Healthy donor plasma and whole blood was obtained from Research Blood Components. Sperm sample of a 39-year-old donor was obtained from Cryos International. To our knowledge, donors were unrelated and samples were deidentified before being sent to us.
Recruitment	Patients were recruited from the Dana-Farber Cancer Institute in Boston, Massachusetts. Healthy donor biological material was obtained from Research Blood Components. Sperm sample was obtained from Cryos International.
Ethics oversight	The IRB of the Dana-Farber Cancer Institute and New York University Grossman School of Medicine approved these protocols. All patients provided written informed consent to allow the collection of blood and/or tumor tissue and analysis of clinical and genetic data for research purposes.

Note that full information on the approval of the study protocol must also be provided in the manuscript.
